# Structure, evolution and functional inference on the *Mildew Locus O* (*MLO*) gene family in three cultivated Cucurbitaceae *spp.*

**DOI:** 10.1186/s12864-015-2325-3

**Published:** 2015-12-29

**Authors:** Paolo Iovieno, Giuseppe Andolfo, Adalgisa Schiavulli, Domenico Catalano, Luigi Ricciardi, Luigi Frusciante, Maria Raffaella Ercolano, Stefano Pavan

**Affiliations:** Department of Agriculture Sciences, University of Naples ‘Federico II’, Via Università 100, 80055 Portici Naples, Italy; Institute of Biosciences and Bioresources, Italian National Council of Research, Via Amendola 165/A, 70126 Bari, Italy; Department of Soil, Plant and Food Science, University of Bari “Aldo Moro”, Via Amendola 165/A, 70126 Bari, Italy

**Keywords:** *MLO*, Cucurbitaceae, Evolution, Powdery mildew, Resistance

## Abstract

**Background:**

The powdery mildew disease affects thousands of plant species and arguably represents the major fungal threat for many Cucurbitaceae crops, including melon (*Cucumis melo* L.), watermelon (*Citrullus lanatus* L.) and zucchini (*Cucurbita pepo* L.). Several studies revealed that specific members of the *Mildew Locus O* (*MLO*) gene family act as powdery mildew susceptibility factors. Indeed, their inactivation, as the result of gene knock-out or knock-down, is associated with a peculiar form of resistance, referred to as *mlo* resistance.

**Results:**

We exploited recently available genomic information to provide a comprehensive overview of the *MLO* gene family in Cucurbitaceae. We report the identification of 16 *MLO* homologs in *C. melo*, 14 in *C. lanatus* and 18 in *C. pepo* genomes. Bioinformatic treatment of data allowed phylogenetic inference and the prediction of several ortholog pairs and groups. Comparison with functionally characterized *MLO* genes and, in *C. lanatus*, gene expression analysis, resulted in the detection of candidate powdery mildew susceptibility factors. We identified a series of conserved amino acid residues and motifs that are likely to play a major role for the function of MLO proteins. Finally, we performed a codon-based evolutionary analysis indicating a general high level of purifying selection in the three Cucurbitaceae *MLO* gene families, and the occurrence of regions under diversifying selection in candidate susceptibility factors.

**Conclusions:**

Results of this study may help to address further biological questions concerning the evolution and function of *MLO* genes*.* Moreover, data reported here could be conveniently used by breeding research, aiming to select powdery mildew resistant cultivars in Cucurbitaceae.

**Electronic supplementary material:**

The online version of this article (doi:10.1186/s12864-015-2325-3) contains supplementary material, which is available to authorized users.

## Background

The *Mildew Locus O* (*MLO*) gene family encodes for plant-specific proteins structurally related to metazoan G-protein coupled receptors (GPCRs), harbouring seven transmembrane domains and a calmodulin-binding domain that is likely implicated in the perception of calcium-dependent stimuli [1, 2]. Genome-wide studies characterized a number of homologs varying from 12 to 19 in the *MLO* gene families of Arabidopsis, grapevine, rice, peach, woodland strawberry and tobacco [1, 3–6].

Specific *MLO* homologs act as susceptibility factors towards fungi causing the powdery mildew (PM) disease, affecting thousands of plant species and representing a major threat in many agricultural settings [7]. Indeed, their inactivation, resulting from gene knock-out or knock-down, leads to a peculiar form of resistance, referred to as *mlo* resistance, based on the enhancement of pre-penetrative defence responses [2, 8, 9]. *mlo* resistance was first reported to occur in barley, following of loss-of-function mutations of the *HvMLO* gene [10]. More recently, knocking-out or knocking-down of specific *MLO* genes was shown to lead to PM resistance in several other plant species, namely Arabidopsis, tomato, pea, pepper and wheat [11–16]. Pleiotropic effects associated with *mlo* resistance do not seem so severe to compromise its practical exploitation [9, 17]. Therefore, it has been suggested to selectively target *MLO* genes of cultivated species as a general breeding strategy against PM [9]. In contrast with other kind of immunities, such as those conferred by plant resistance genes (R-genes), a body of evidence indicates that *mlo* resistance is characterized by broad-spectrum effectiveness and durability. For example, barley *mlo* resistance is effective against all known isolates of the PM fungus *Blumeria graminis* f. sp. *hordei*, and is successfully employed in barley cultivars since 1979 [18]. Similarly, pea *er1* PM resistance, due to loss of function mutations of the *PsMLO1* gene, was first reported in 1948 and is the only resistance source worldwide used for breeding purposes [19, 20].

Previous phylogenetic studies grouped MLO proteins in six clades [1]. Available scientific literature indicates that the clades referred to as IV and V, displaying peculiar molecular features, include a few homologs playing a major role in PM susceptibility in monocots and dicots, respectively [21, 22]. Moreover, corresponding *MLO* genes are strongly upregulated upon challenge with PM fungi (e.g. [5, 12, 13]. Taken together, this body of evidence indicates that phylogenetic inference, multiple alignment and gene expression studies are all important tools to identify, across cultivated species, novel *MLO* homologs determining the outcome of the interaction with PM fungi.

PM caused by the fungal species *Golovinomyces cichoracearum* and *Podosphaera xanthii* is considered to be one of the main biotic threat for the cultivation of species of the Cucurbitaceae family [23–26]. Melon (*Cucumis melo* L.), watermelon (*Citrullus lanatus* L.) and zucchini (*Cucurbita pepo* L.) are three important horticultural Cucurbitaceae species, grown throughout the world and of great interest for nutritional properties as well as for economic importance. Recently, genome sequence of these three species was released [27, 28], (https://cucurbigene.upv.es/genome-v3.2/). Several putative disease resistance genes were identified in the melon genome. Among them, 15 genes were classified as transmembrane receptors and found to be homologous to barley *HvMLO* [27]. No member of the watermelon and zucchini *MLO* gene families was isolated so far.

Here, we used melon, watermelon and zucchini genomic resources to characterize the corresponding *MLO* gene families, and gain information with respect to their structure, evolutionary history and function. Among other things, this could result extremely useful for practical breeding activities aiming to select PM resistant cultivars in these species.

## Results

### Characterization of the *MLO* gene family in *C. melo, C. lanatus* and *C. pepo*

In order to identify MLO proteins in melon, a BLASTp analysis against the publicly available genomic database of *C.melo* was performed. This yielded 20 significant matches, each corresponding to a MLO-like hit. Two of the newly identified CmMLO proteins (named MELO3C022486 and MELO3C000169 in the database) resulted to be identical to different regions of the melon homolog CmMLO1, previously characterized [29]. We decided to discard these sequences and keep the corresponding *CmMLO1* gene for further analysis since its sequence and expression was experimentally validated by Cheng et al. [29]. *CmMLO* genes corresponding to the newly identified proteins were named from *CmMLO2* to *CmMLO16* (Table 1).

**Table 1:** Features of the *C. melo MLO* gene family

*MLO* name	Locus name	Scaffold/ Conting	Starting position (Mb)	Linkage Group	Clade	Introns	Length (aa)
*CmMLO1*	ACX55085.1	-	-	-	II	0	514
*CmMLO2*	MELO3C005038	4	8.64	12	-	4	199
*CmMLO3*	MELO3C005044	4	8.77	12	V	13	584
*CmMLO4*	MELO3C007979	7	6.58	8	III	14	586
*CmMLO5t1*	MELO3C012438t1	16	5.19	10	V	14	574
*CmMLO5t2*	MELO3C012438t2	16	5.19	10	V	14	563
*CmMLO6*	MELO3C013709	21	0.16	6	III	14	576
*CmMLO7*	MELO3C013761	21	0.52	6	-	14	484
*CmMLO8t1*	MELO3C016709t1	29	1.54	7	I	13	505
*CmMLO8t2*	MELO3C016709t2	29	1.54	7	I	14	565
*CmMLO9*	MELO3C019435	38	0.97	n.a.	I	14	560
*CmMLO10t1*	MELO3C021515t1	48	0.39	9	I	16	557
*CmMLO10t2*	MELO3C021515t2	48	0.39	9	I	15	588
*CmMLO11*	MELO3C023219	59	0.34	11	II	11	469
*CmMLO12*	MELO3C025761	82	0.98	11	V	12	441
*CmMLO13*	MELO3C026525	96	0.27	3	III	14	546
*CmMLO14*	MELO3C025760	82	0.98	11	-	0	80
*CmMLO15*	MELO3C005037	4	8.63	12	-	0	147
*CmMLO16*	MELO3C007252	7	1.74	8	-	1	150

Information reported in the table was extracted by the GenBank genomic database (for *CmMLO1*) and the Melonomics genomic database (for the other *CmMLO* homologs)

Database search revealed the occurrence of additional splicing variants for the *C. melo* homologs *CmMLO5*, *CmMLO8* and *CmMLO10* (Table 1). With the exception of *CmMLO9*, chromosomal localizations of *CmMLO* genes were inferred based on the presence, on the same scaffold, of single nucleotide polymorphism (SNP) markers mapped to melon linkage groups [27] (Additional file 1). Predicted intron/exon structure and corresponding protein amino acid length were also obtained by database search (Table 1).

A similar *in silico* approach allowed the identification of members of the MLO protein family in *C. lanatus*. BLASTp of melon MLO protein sequences against the Cucurbit genomics database resulted in the identification of 14 significant matches annotated as MLO-like proteins. Corresponding genes were named from *ClMLO1* to *ClMLO14*. For all of them, it was possible to determine chromosomal localization and intron/exon boundaries (Table 2, Additional file 1).

**Table 2:** Features of the *C. lanatus MLO* gene family

*MLO* name	Locus name	Chromosome	Starting position (Mb)	Clade	Introns	Length (aa)	*C. melo* orthologs
*ClMLO1*	Cla002071	2	17.23	VII	12	532	
*ClMLO2*	Cla005044	3	2.43	V	13	586	*CmMLO3*
*ClMLO3*	Cla005046	3	2.49	VI	12	534	
*ClMLO4*	Cla006975	6	0.51	II	11	514	*CmMLO1*
*ClMLO5*	Cla008753	2	31.28	V	13	604	*CmMLO12*
*ClMLO6*	Cla008904	10	1.65	I	12	493	*CmMLO7*
*ClMLO7*	Cla008957	10	2.11	III	14	582	*CmMLO6*
*ClMLO8*	Cla009651	1	31.83	VII	13	501	
*ClMLO9*	Cla010381	9	31.04	I	14	553	*CmMLO10*
*ClMLO10*	Cla013018	5	9.93	I	11	512	*CmMLO9*
*ClMLO11*	Cla014358	1	30.21	III	13	563	*CmMLO4*
*ClMLO12*	Cla020573	5	28.57	V	14	561	*CmMLO5*
*ClMLO13*	Cla021922	8	18.59	III	13	523	*CmMLO13*
*ClMLO14*	Cla023394	11	20.29	I	12	500	*CmMLO8*

Information on single *MLO* homologs was inferred by the Cucurbit Genomic Database. Relations of orthology with *C. melo MLO* genes, predicted by the present study, are also reported

*Cucurbita pepo* homologs harbouring a typical MLO domain were identified by using an in-house-built pipeline described in the materials and methods section. These were designated sequentially from *CpMLO1* to *CpMLO18* based on the length of their putatively encoded protein (Table 3). Localization of the corresponding genes on *C. pepo* scaffolds was predicted by means of data available at the Cucurbigene genomic database (Table 3).

**Table 3:** Features of the *C. pepo MLO* gene family

*MLO* name	Scaffold	Starting position (Mb)	Clade	Introns	Lenght (aa)	*C. melo* orthologs	*C. lanatus* orthologs
*CpMLO1*	74	0.51	-	1	121	-	-
*CpMLO2*	9	2.94	-	0	142	-	-
*CpMLO3*	74	0.51	-	5	272	*CmMLO1*	*ClMLO4*
*CpMLO4*	70	0.23	-	4	331	*-*	*-*
*CpMLO5*	1	1.65	-	6	334	*-*	*-*
*CpMLO6*	20	1.87	-	6	358	*-*	*-*
*CpMLO7*	42	1.43	-	7	384	*-*	*-*
*CpMLO8*	30	2.65	-	8	394	*-*	*-*
*CpMLO9*	14	2.69	V	8	441	*CmMLO5*	*ClMLO12*
*CpMLO10*	4	2.47	V	8	442	*CmMLO12*	*ClMLO5*
*CpMLO11*	13	1.68	III	9	444	*CmMLO4*	*ClMLO11*
*CpMLO12*	52	1.34	VI	7	458	*-*	*ClMLO3*
*CpMLO13*	22	2.59	V	9	492	*CmMLO12*	*ClMLO5*
*CpMLO14*	12	0.20	II	11	495	*CmMLO1*	*ClMLO4*
*CpMLO15*	37	1.77	I	10	499	*CmMLO7*	*ClMLO6*
*CpMLO16*	52	1.30	V	11	596	*CmMLO3*	*ClMLO2*
*CpMLO17*	29	2.20	III	13	625	*CmMLO4*	*ClMLO11*
*CpMLO18*	8	3.73	III	12	904	*CmMLO6*	*ClMLO7*

Information on single *MLO* homologs was inferred by the Cucurbigene genomic database. Relations of orthology with *C. melo* and *C. lanatus MLO* genes, predicted by the present study, are also reported

### Phylogenetic analysis

**Fig. 1:**
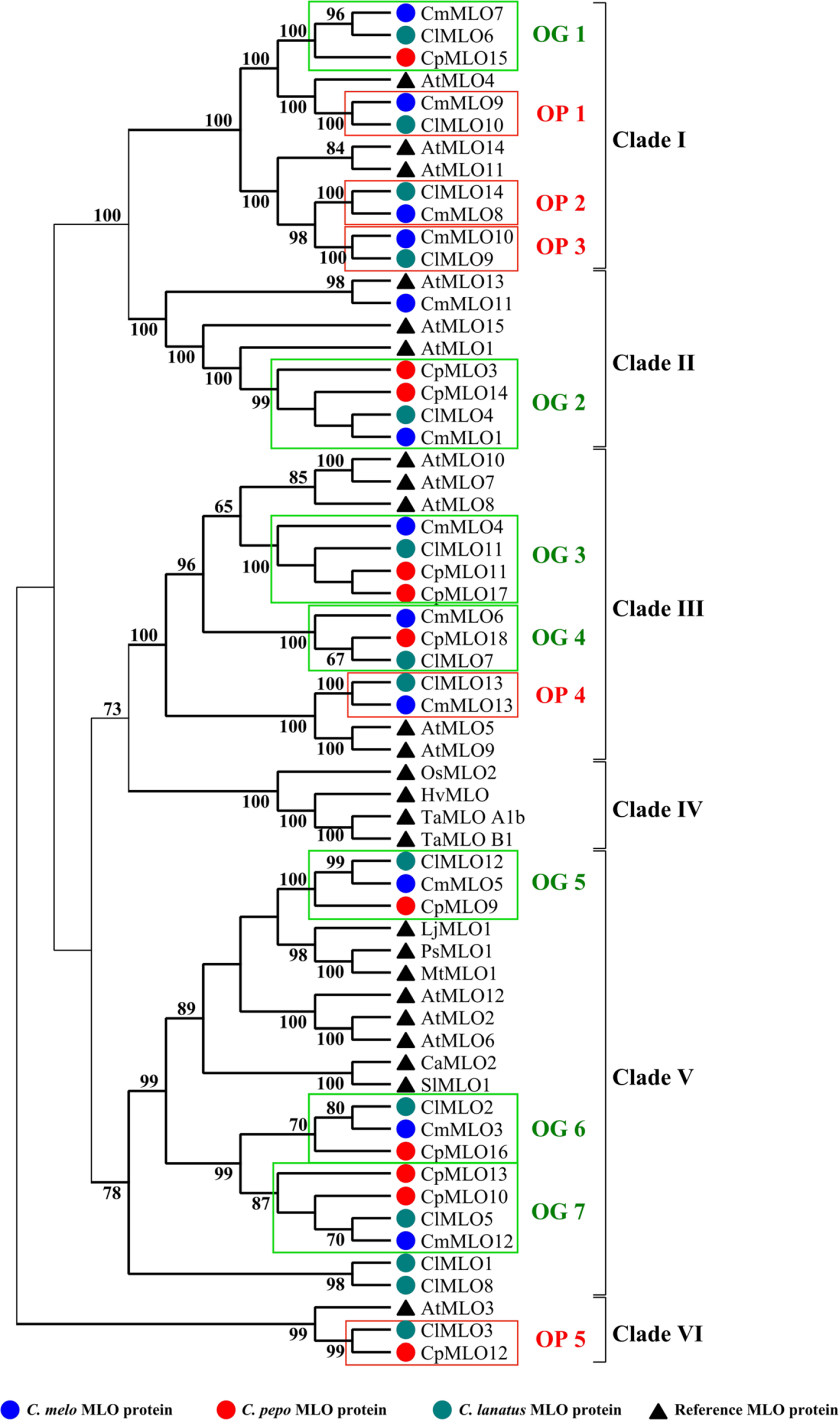
Maximum likelihood phylogenetic tree of Cucurbitaceae MLO proteins. The tree, obtained by the Whelan and Goldman model, includes 37 Cucurbitaceae homologs harbouring at least 50 % of the MLO-Pfam domain and 24 reference MLO proteins already characterized in other species. Clades were numerated with the Roman numerals from I to VI, according to Devoto et al. 2003 [1] and based on the position of Arabidopsis and monocot MLO homologs. The tree was drawn to scale, with branch lengths proportional to the number of substitutions per site. Bootstrap values higher than 60 (out of 100 replicates), are indicated above the branches. Green boxes highlight seven putative orthologous groups (OG) of Cucurbitaceae MLO proteins with a bootstrap support ≥ 70, positioned in four (I, II, III and V) phylogenetic clades; red boxes indicate five orthologous pairs (OP) with bootstrap support ≥ 99

The MLO-Pfam domain of *C. melo*, *C. lanatus* and *C. pepo* MLO proteins was used to infer phylogenetic distances between them and with respect to MLO homologs of other plant species (Fig. 1). In total, 61 MLO proteins (pairwise average identity: 38.3 %) collapsed in six phylogenetic clades (bootstrap index ≥78) (Fig. 1). These were designated with the Roman numerals from I to VI, based on the position of Arabidopsis and monocot MLO homologs, according to the previous study of [1].

Strong bootstrap support was found for the presence of a common ancestor in the evolutionary history of clades I and II (Fig. 1). Clade I includes 12 MLO proteins, three of which are annotated in Arabidopsis (AtMLO4, AtMLO11 and AtMLO14), four in *C. melo* (CmMLO7, CmMLO8, CmMLO9 and CmMLO10), four in *C. lanatus* (ClMLO6, ClMLO9, ClMLO10 and ClMLO14) and one in *C. pepo* (CpMLO15). Clade II groups eight MLO homologs, annotated in Arabidopsis (AtMLO1, AtMLO13 and AtMLO15), *C. melo* (CmMLO1 and CmMLO11), *C. lanatus* (ClMLO4) and *C. pepo* (CpMLO3 and CpMLO14).

Phylogenetic data also provided evidence for a common ancestor originating clades III and IV (Fig. 1). Clade III includes nine Cucurbitaceae homologs (CmMLO4, CmMLO6, CmMLO13, CpMLO11, CpMLO17, CpMLO18, ClMLO7, CmMLO11, and ClMLO13) together with five Arabidopsis proteins (AtMLO5, AtMLO7, AtMLO8, AtMLO9 and AtMLO10). Clade IV contains MLO proteins from monocot species only.

Three melon (CmMLO3, CmMLO5 and CmMLO12), four zucchini (CpMLO9, CpMLO10, CpMLO13 and CpMLO16) and five watermelon proteins (ClMLO1, ClMLO2, ClMLO5, ClMLO8 and ClMLO12) cluster together in the phylogenetic clade V, including all the dicot MLO homologs experimentally shown to act as PM susceptibility factors [11–15]. Finally, clade VI is located on an ancestral position, lacking *C. melo* homologs and harbouring only three proteins (AtMLO3, ClMLO3 and CpMLO12).

A further phylogenetic analysis was performed using nucleotide sequences (including introns) of the *C. melo*, *C. lanatus* and *C. pepo* gene families (Additional file 2). This did not provide clear evidence of recent gene duplication events, as the rate of nucleotide identity of monophyletic *MLO* pairs resulted to be very low. For each of the three species, the highest levels of pairwise nucleotide identity concern the pairs *CmMLO4*-*CmMLO6* (49.8 %), *ClMLO9*-*ClMLO14* (47 %) and *CpMLO3*-*CpMLO14* (62.7 %) (Additional file 2).

### Orthology between Cucurbitaceae *MLO* families

**Fig. 2:**
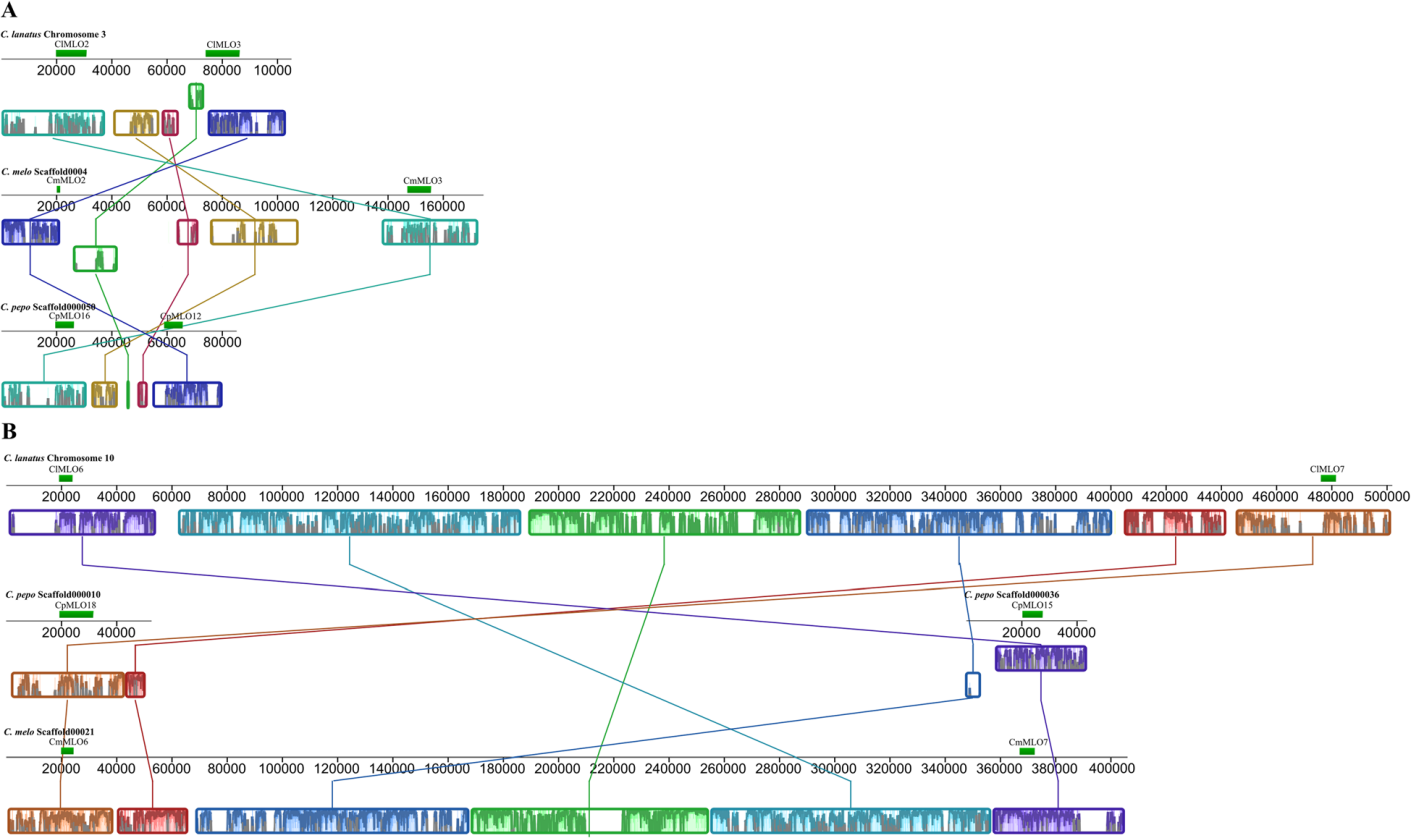
Identification of putative Cucurbitaceae *MLO* orthologs in synthenic genomic regions. *C. lanatus*, *C. melo* and *C. pepo MLO* loci are indicated with solid green boxes. Collinear blocks are labelled with the same colour and connected by lines. Block boundaries indicate breakpoints of genome rearrangements. Panel **a**) refers to the triads *ClMLO2*, *CmMLO3*, *CpMLO16* and *ClMLO3, ClMLO2* and *CpMLO12*; Panel **b**) refers to the triads *ClMLO6*, *CpMLO15*, *CmMLO7* and *ClMLO7*, *CpMLO18, CmMLO6*

Phylogenetic analysis supported the identification of five putative ortholog pairs (OP1-OP5; bootstrap index ≥99) and seven putative ortholog groups (OG1-OG7; bootstrap value ≥70) including *C. melo, C. lanatus* and *C. pepo* MLO homologs (Fig. 1). The putative ortholog groups 2, 3 and 7 included three *C. pepo* paralog pairs, CpMLO3-CpMLO14, CpMLO11-CpMLO17 and CpMLO10-CpMLO13, respectively (bootstrap value ≥87). In order to provide further evidence for the identification of Cucurbitaceae orthologs, we performed multiple genome alignments of *C. melo*, *C. lanatus* and *C. pepo* genomic regions harbouring *MLO* genes. We reasoned that, in case of orthology, these regions, sharing a common ancestry, would have displayed high level of syntheny. Remarkably, all the *MLO* homologs clustering in the same ortholog group or pair were found to be located in syntenic genomic regions (Fig. 2, Additional file 3, 4, 5 and 6), thus substantiating the predictions resulting from the phylogenetic study.

### Characterization of conserved amino acids and motifs

To examine sequence features of melon, watermelon and zucchini MLO proteins, a full-length multiple sequence alignment of 48 Cucurbitaceae proteins was performed. For comparative purposes, we completed the dataset with 12 MLO proteins from 10 different plant species (*Solanum lycopersicum*, *Capsicum annum*, *Nicotiana tabacum*, *Medicago truncatula*, *Pisum sativum, Arabidopsis thaliana*, *Lotus japonicus*, *Triticum aestivum*, *Hordeum vulgare* and *Oryza sativa*), known to play a major role in the interaction with PM fungi. Aligned regions of the Cucurbitaceae MLO homologs showed very high conservation levels (>95 %) with respect to the 30 amino acid residues previously indicated as invariable throughout the whole MLO protein family [30] (Additional file 7). Moreover, we found other 13 residues highly conserved (>95 %) in the Cucurbitaceae MLO families as well as in the other MLO homologs used for multiple alignment. These are distributed in the extracellular N-terminal region (T^83^), the 1^st^ intracellular loop (T^85^,W^86^, A^89^and V^91^), the 2^nd^ transmembrane domain (L^162^, K^168^, K^170^ and L^181^), the 1^st^ extracellular loop (P^219^), the 2^nd^ intracellular loop (K^339^ and W^347^) and the 3^rd^ intracellular loop (D^594^) (Additional file 7).

Analysis of MLO proteins belonging to clade V revealed the presence of 125 conserved (>90 %) residues in common between Cucurbitaceae and other species (Additional file 7), thus indicating they could specifically play a role in the response to PM fungi. Moreover, 25 patterns of consecutive conserved amino acids (motifs), distributed along all the known domains of MLO proteins and ranging in size from 2 to 11 residues, were identified (Additional file 7).

### Selection pressure acting on Cucurbitaceae *MLO* gene families

The dissimilarity level between the non-synonymous substitution (dN) and synonymous substitution (dS) values was used to infer the direction and magnitude of natural selection acting on *MLO* genes in *C. melo, C. lanatus* and *C. pepo*. Neutrality tests, performed on the *MLO* gene family of each of the three Cucurbitaceae species, yielded average δ (dN-dS) and ω (dN/dS) values ranging from −23.89 to −17.58 and from 0.29 to 0.41, respectively (Table 4). This indicates that negative selection has been acting against extreme polymorphic variants. In particular, the *MLO* families of *C. lanatus* and *C. melo*, whose members have average sequences identity of 42.6 % and 46.8 %, respectively, appear to be subjected to very high negative selection pressures (δ = −23.89; ω = 0.31 and δ = −22.92; ω = 0.29). The *C. pepo MLO* family, including members with average identity of 33.4 %, is characterized by a softer level of negative selection (δ = −17.54; ω = 0.41). Single codon analysis highlighted the presence of 36, 134 and 127 negatively selected sites in the *C. pepo, C. lanatus* and *C. melo MLO* families, respectively. With respect to other phylogenetic clades, clade V displays lower δ (−27.73) and ω (0.27) values (Table 4), thus suggesting that it is subjected to a larger number of selective constraints.

**Table 4:** Average nucleotide diversity (π) and non synonymous to synonymous substitutions mean dissimilarity analysis

Aligned coding sequences	*MLO* coding sequences (No)	π	δ	ω
*C. pepo MLO* family	11	0.21	−17.54	0.41
*C. lanatus MLO* family	14	0.33	−23.89	0.31
*C. melo MLO* family	12	0.34	−22.92	0.29
Cucurbitaceae *MLO* clade I	9	0.31	−18.42	0.31
Cucurbitaceae *MLO* clade II	4	0.09	−10.47	0.34
Cucurbitaceae *MLO* clade III	9	0.19	−23.65	0.32
Cucurbitaceae *MLO* clade V	12	0.22	−27.73	0.27
Cucurbitaceae *MLO* clade VI	2	0.32	−6.53	-

All the values of δ (=d_N_-d_S_) and ω (=d_N_/d_S_) reported in the table, estimated from different alignments of *MLO* coding sequences, were calculated using the Nei-Gojobori and SLAC methods, respectively, and are significant (*p* < 0.05 and *p* < 0.1) over the null hypothesis of strict-neutrality (d_N_ = d_S_;d_N_/d_S_ = 1) in favour of the alternative hypotheses of positive (d_N_ > d_S_;d_N_/d_S_ > 1) or negative (d_N_ < d_S_; d_N_/d_S_ < 1) selections

**Fig. 3:**
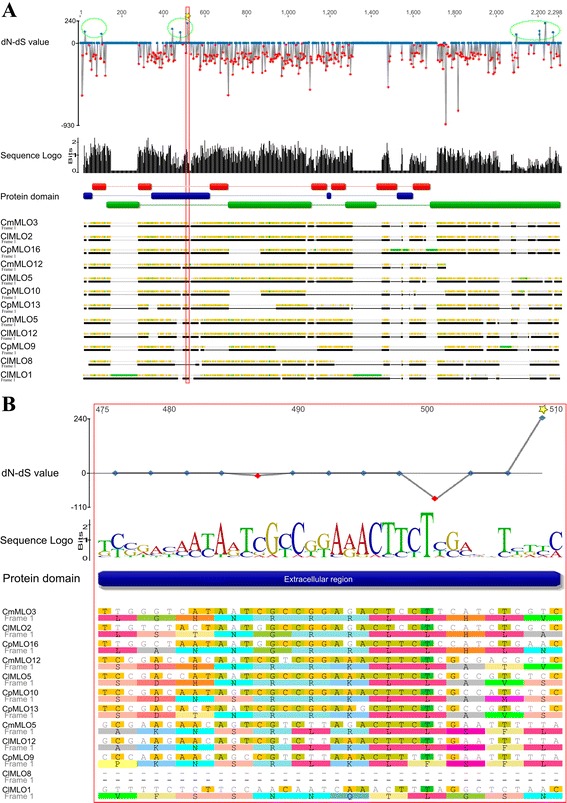
Direction and magnitude of natural selection acting on Cucurbitaceae clade V *MLO* homologs. **a**) Codon-based dN-dS values and sequence logo indicating nucleotide conservation along the alignment of Cucurbitaceae Clade V *MLO* coding sequences. Blue and red dots indicate positive and negative values, respectively. Green circles indicate protein regions containing codons predicted to be subjected to positive selection, and the star indicates a codon characterized by a significant p-value (*p* < 0.1) for positive selection (in position 252 of the alignment). Aligned sequences are also indicated, together with gene regions predicted to encode for extracellular, transmembrane and intracellular protein regions (indicated with blue, red and green bars, respectively). **b**) Close-up view on the gene region containing the codon in position 252 of the alignment

**Fig. 4:**
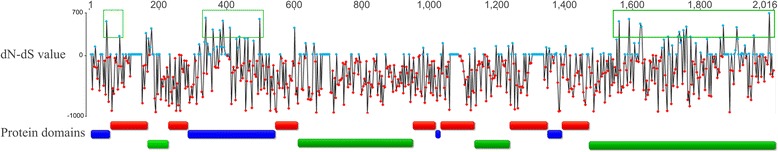
Direction and magnitude of natural selection acting on Rosaceae clade V *MLO* homologs. Blue and red dots indicate positive and negative dN-dS values, respectively. Gene regions encoding for extracellular, transmembrane and intracellular protein regions are indicated with the blue, red and green bars, respectively). Green boxes indicate protein regions in the N-terminus, first transmembrane domain, first extracellular loop and intracellular C-terminus containing codons predicted to evolve through positive selection

Interestingly, single codon evolutionary analysis of clade V homologs also revealed the presence of three protein regions, located in the 1^st^ transmembrane domain, the 1^st^ extracellular loop and the intracellular C-terminus domain, harbouring several residues predicted to be under positive selection pressure. Despite the low number of sequences used for the analysis, one of them (in position 252 of the alignment in Additional file 7) is characterized by a p-value <0.1 (considered to be a stringent significant threshold for single likelihood ancestor counting (SLAC) evolutionary analysis) (Fig. 3). In order to validate this finding, we analysed the direction and magnitude of natural selection acting on the Rosaceae clade V *MLO* homologs reported by [5]. Notably, this resulted in the identification of several codons characterized by high dN-dS values and encoding for residues also positioned in the protein 1^st^ transmembrane domain, 1^st^ extracellular loop and intracellular C-terminus (Fig. 4).

### *C. lanatus CmMLO12* is upregulated upon PM challenge

**Fig. 5:**
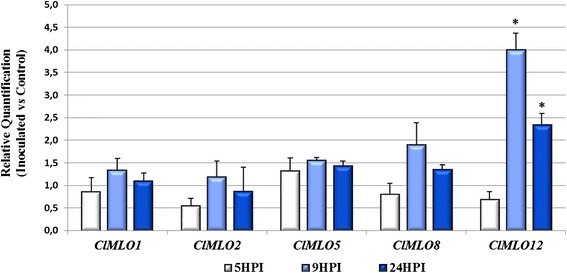
Transcriptional variation of *C. lanatus* clade V *MLO* genes in response to PM challenge. Data refer to leaves of the watermelon cultivar ‘Sugar Baby’, sampled at three different time points 5, 9 and 24 h post inoculation (HPI) with the PM fungus *Podosphaera xanthii*. For each time point, relative expression levels are normalized with respect to the housekeeping gene β-Actin and compared to non-inoculated controls. Standard error bars refer to a number of biological replicates ranging from three to four. Significant differences between the means were inferred using the Student’s *t* test for each time point (*p < 0.05)

A distinctive feature of *MLO* susceptibility genes is their up-regulation upon PM challenge. Therefore, we quantified relative expression levels of clade V watermelon *MLO* genes (*ClMLO1, ClMLO2, ClMLO5, ClMLO8* and *ClMLO12)* in leaves artificially inoculated with the PM fungus *Podosphaera xanthii* and in non-inoculated controls. No significant difference was detected for *ClMLO1*, *ClMLO2*, *ClMLO5* and *ClMLO8*. Notably, strong up-regulations was observed for *ClMLO12* at the time points corresponding to 9 and 24 h after inoculation, indicating that it is a pathogen-responsive gene (Fig. 5).

## Discussion

In this study, we exploited available genomic resources to characterize the *MLO* gene family in cultivated Cucurbitaceae. A total of 16, 14 and 18 *MLO*-like gene sequences were identified in the genomes of *C. melo*, *C. lanatus* and *C. pepo*, respectively. This is consistent with the results of previous genome-wide surveys, reporting the presence of a number of *MLO* homologs variable from 15 and 19 [1, 3, 5, 6]. All the predicted *C. lanatus* MLO proteins resulted to have amino acid lengths comparable to those of Arabidopsis AtMLO homologs, ranging from 460 to 593 residues [1]. In contrast, predicted length of some of the *C. melo* and *C. pepo* MLO homologs resulted be markedly shorter or, in one case, longer than these limits (Tables 1, 2 and 3), possibly due to incorrect prediction.

Information on chromosome/scaffold localization of *C. melo* and *C. lanatus MLO* homologs revealed that most of them occur as singletons, thus indicating they have been mainly originated by segmental duplication events. This is consistent with previous investigations on the evolution of the *MLO* gene family in Rosaceae species and in soybean [5, 31]. Nonetheless, for each of the two species, three pairs of physically close homologs were found (CmMLO2-3, CmMLO6-7, CmMLO12-14, ClMLO2-3, ClMLO6-7, ClMLO8-11) (Additional file 1), which are likely the result of tandem duplication. None of the duplication event in the *C. pepo*, *C. lanatus* and *C. melo* genomes appeared to have occurred recently, as resulting from the computational analysis performed in this work indicating a low rate of nucleotide identity between monophyletic *MLO* pairs (Additional file 2).

Relations of orthology between Cucurbitaceae *MLO* homologs were inferred based on both phylogenetic relatedness and localization in microsynthenic regions. This resulted in the prediction of five ortholog pairs and seven ortholog groups (Fig. 1). Importantly, in support of our conclusions, all the *C. lanatus*/*C. melo* ortholog pairs predicted in this study are positioned in macrosynthenic genomic regions between the two species, described by Guo et al. [28] (Additional file 8). In three cases, ortholog groups were found to contain 2 homologs from *C. pepo* (Fig. 1). Possibly, these might represent co-orthologs relative to *C. melo* and *C. lanatus* proteins falling in the same group, which originated from duplication during the evolutionary history of *C. pepo* genome.

Phylogenetic analysis allowed to assign Cucurbitaceae MLO proteins to the five evolutionary clades previously reported to include homologs from dicotyledonous species (Fig. 1). Three melon, three watermelon and four zucchini MLO homologs were found to cluster together in the phylogenetic clade V, containing all the dicot isoforms functionally associated with PM susceptibility. Moreover, we demonstrated that watermelon *ClMLO12* is up-regulated during pathogenesis, a feature which is shared by other PM susceptibility factors (e.g. [5, 12, 13]). Thus, with our study we provide information on targets of future activities addressed to the inactivation of PM susceptibility genes and, thus, the selection of resistant genotypes. Several approaches of reverse genetics are available to breeders interested in *MLO* gene knock-out or knock down, such as those based on RNA interference (RNAi), targeted induced local lesions in genomes (TILLING), transcription activator-like effector nucleases (TALEN), zinc finger nucleases (ZFNs) and clustered regularly interspaced short palindromic repeat (CRISPR) technology [32–34]. Noteworthy, TALEN and CRISPR technology have been recently successfully used to introduce *mlo* resistance in bread wheat, as reported by Wang et al. [16].

We performed a multiple alignment of a large dataset MLO proteins, aiming to detect amino acid residues and motifs that, being highly conserved, are predicted to play a major role for protein function. As expected, a very high conservation level was found with respect to the 30 amino acids previously shown to be invariable in a panel of 38 selected MLO sequences [30]. In addition, we detected other 13 highly conserved residues (Additional file 7). Alignment, restricted to isoforms known to act as PM susceptibility factors and clade V Cucurbitaceae MLO homologs, also revealed a series of conserved amino acid residues and motifs scattered in several protein domains (Additional file 7). These might be specifically important for the function of isoforms associated with PM susceptibility. With this respect, our study complements the previous work of Panstruga [35], reporting the identification, in the MLO protein first extracellular region, second and third intracellular regions and the cytoplasmic C-terminus, of a series of amino acids specifically conserved in putative orthologs of barley HvMLO.

In line with the identification of several conserved residues, tests addressed to infer evolutionary forces acting on Cucurbitaceae *MLO* homologs suggested a general high level of negative selection (Table 4), thus pointing to the presence of a series of constraints required for protein function. Interestingly, single codon analysis of Clade V Cucurbitaceae and Rosaceae *MLO* homologs also highlighted the occurrence of three protein regions that are likely under positive selection pressure (Fig. 3 and 4). Positive selection is a common phenomenon which drives plant/pathogen co-evolution, in accordance to an “arms race” model [36]. For example, positive selection been shown to occur for solvent-exposed residues of plant resistance (*R*) genes, as it provides an advantage in pathogen recognition [37]. It might be tempting to speculate that positively selected *MLO* residues located in the first extracellular loop or in the intracellular C-terminus might also be implicated in pathogen sensing. However, the role of MLO proteins in the interaction with PM fungi is still elusive to date, and no molecular interaction between MLO proteins and pathogen effectors has been reported so far.

## Conclusions

In the present study, we carried out a genome-wide characterization of the *MLO* gene families in three economically important Cucurbitaceae species. Importantly, our results also lay a foundation for future breeding activities aimed at introducing PM resistance.

## Methods

### Database search in *C. melo, C. lanatus* and *C. pepo* genomes

In order to retrieve predicted melon *MLO* genes and MLO proteins, a BLASTp search (e-value < 1e-5) was carried out against the CM3.5.MELO.3C protein repositories publicly available at the Melonomics melon genomic database (https://melonomics.net). Homologs were assigned to melon linkage groups based on the reference map position of SNP markers belonging to the same scaffold.

Watermelon MLO-like protein sequences were isolated by blasting each of the 18 melon protein sequences against the watermelon_v1.pep repository available at the Cucurbit Genomic Database (http://www.icugi.org/cgi-bin/ICuGI/index.cgi). Information on chromosomal localization of each of the corresponding genes was available at the same database.

Aiming to the characterization of the *MLO* gene family in zucchini, a bioinformatic pipeline was developed in house. We used available *Cucurbita pepo* unigenes (http://www.icugi.org/cgi-bin/ICuGI/misc/download.cgi) for a BLASTn analysis against *C. pepo* scaffolds (https://cucurbigene.upv.es/genome-v3.2/), in order to discover *C. pepo* scaffolds harbouring *MLO* loci. A subsequent MLO prediction on *C. pepo* candidate scaffolds led to the identification of 18 *MLO* genes. Cds and encoded sequences of zucchini *MLO*s were predicted using the GeneScan software (http://genes.mit.edu/GENSCAN.html).

### Multiple sequence alignments and phylogenetic analysis

Sequence similarities were determined performing a MUSCLE (multiple sequence comparison by log- expectation) multiple alignment [38] using the conserved MLO domain sequence of the PFAM database (ID: PF03094) as input. For the three melon MLO homologs for which two transcripts were identified in database, the longest was used for the analysis. Phylogenetic analysis was performed by using newly identified Cucurbitaceae MLO homologs containing at least 50 % of the full-length MLO domain (12 from *C. melo*, 14 from *C. lanatus* and 11 from *C. pepo*). The dataset was completed with the whole Arabidopsis MLO protein family and the following proteins previously shown to act as PM susceptibility factors: pea PsMLO1, barley HvMLO, rice OsMLO2, pepper CaMLO2, tomato SlMLO1, barrel clover MtMLO1, lotus LjMLO1, and wheat TaMLO1_A1b and TaMLO_B1. All of these sequences were extracted from the NCBI database (http://www.ncbi.nlm.nih.gov). Evolutionary relationships between MLO proteins were inferred using the maximum likelihood method based on the Whelan and Goldman model [39], using the MEGA6 software (http://www.megasoftware.net) [40]. The bootstrap consensus tree, obtained from 100 replicates, was taken to represent the MLO family phylogenetic history [41]. To the characterization of conserved amino acids and motifs a full-length multiple alignment was conducted using the 48 Cucurbitaceae MLO proteins identified in this study and 12 reference MLO proteins characterized in other species (Additional file 4).

### Evolution rates at codon sites

Evolutionary forces acting on *MLO* homologs, in Cucurbitaceae and Rosaceae families, were investigated by determining two parameters based on the number of non synonymous and synonymous substitutions per non synonymous and synonymous site (dN and dS, respectively), indicated as δ (dN-dS) and ω (dN/dS). Tests were conducted to estimate the evolution of each codon: positive (dN > dS); neutral (dN = dS); and negative (dN < dS). The variance of the difference was computed using the bootstrap method (1000 replicates). Analyses were conducted using the Nei-Gojobori method [42] implemented in the MEGA6 software [40]. All *MLO* coding DNA sequences were aligned using ClustalW 1.74 [43] and positions with less than 80 % site coverage were eliminated from the analysis. To clearly depict the proportion of sites under selection, an evolutionary fingerprint analysis was carried out using the implemented SLAC algorithm in the Datamonkey server at the default value [44]. Pressure selection analysis were performed on the same sequences subset of phylogenetic analysis.

### Prediction of *MLO* orthologs

Relationships of orthology between Cucurbitaceae *MLO* genes were inferred based on phylogenetic distance. Furthermore, synthenic chromosomal regions containing putative *MLO* orthologs were searched in the genomes of melon, watermelon and zucchini by using the MAUVE (Multiple Alignment of Conserved Genomic Sequence with Rearrangements) software package [45]. To determine a reasonable value for the Min Locally Collinear Blocks (LCBs), we performed an initial alignment at the default value and then used the LCB weight slider in the MAUVE graphical user interface (GUI) to fix parameters empirically eliminating all spurious rearrangements. Sequences were then realigned using the manually determined weight value.

### Inference on *MLO* genes duplication events

To identify duplicated homolog pairs in the *C. melo, C. lanatus* and *C. pepo MLO* gene families, we run a phylogenetic analysis using nucleotide sequences (including introns), using the Maximum Likelihood method and applying General Time Reversible model. We defined a gene duplication according to the following criteria: (1) clade bootstrap index >70, (2) alignable nucleotide sequence identity ≥70 % (3) putative recent duplications were also filtered for physical chromosome/scaffold co-localization and (4) only one event of duplication is counted for tightly linked genes. The alignments of these dataset were conducted using ClustalW 1.74 [43]. These criteria are described by Andolfo et al. [46].

### Expression analysis of powdery mildew effector candidates in *Citrullus lanatus*

Plants of *C. lanatus* cv. Sugar Baby were kindly provided by the Semiorto Sementi Seed Company (Sarno, Italy). Inoculation with PM fungus was performed on the third true leaf of 10 cm high plants, by touching with heavily sporulating leaves of spontaneous infected *C. lanatus* cv. Sugar Baby. Leaf samples were collected 5, 9 and 24 h after artificial inoculation with PM fungus *Podosphaera xantii*. Plants touched with healthy leaves at the same time points were used as controls. For each treatment, four biological replicates were sampled. Total RNA was isolated from ground, frozen leaf tissues using the Spectrum^TM^ Plant Total RNA Kit (Sigma-Aldirch). A complete removal of traces of DNA was performed using On-Column DNase I Digest Set (Sigma-Aldirch). RNA quantity and quality were measured spectrophotometrically by the NanoDrop ND-1000 Spectrophotometer (NanoDrop Technologies) and on a denaturing formaldehyde gel. Reverse transcription was performed in a volume of 20 μl using the SuperScript III Reverse Transcriptase kit (Invitrogen) with oligo-dT primers. Resulting cDNAs were diluted (1:20) with autoclaved distilled water and stored at −20 °C until further analysis.

The expression of of *MLO* genes grouping in the phylogenetic clade V (*CmMLO1*, *CmMLO2*, *CmMLO5*, *CmMLO8* and *CmMLO12*) was monitored through qRT-PCR; using the 7900HT Fast RealTime PCR System (Applied Biosystems). Reactions were prepared in a total volume of 12 μL with 6 μL of the 2X SensiFAST™ Probe Hi-ROX Kit (Bioline), 0.4 pmol of target gene primers (Additional file 6) and 4 μL of cDNA template. PCR cycling conditions were as follows: 95 °C for 10 min, followed by 40 cycles of two steps: 95 °C for 15 s and 60 °C for 1 min. A dissociation kinetics analysis was performed after each assay in order to check the specificity of the amplification products. The melting curve programme was set from 60 °C to 95 °C with a 2 % heating rate and a continuous fluorescence measurement. For each time point, relative quantification of gene expression was carried out using the 2^−ΔΔCt^ method [47] with respect to the untreated control sample (calibrator). Primers designed on the housekeeping gene β-Actin (Additional file 9) were used for normalization of the expression levels of the target gene, as it was reported to as a suitable housekeeping by Kong et al. [48]. The Student's *t*-test was used in statistical data analysis procedure for hypothesis testing.

## Availability of supporting data

All the supporting data is included within the article (and its additional files).

## Additional files

Additional file 1: Figure S1. Chromosomal localization of *C. melo* and *C. lanatus MLO* genes. Positions are estimated based on available information on physical localization on melon linkage groups or watermelon scaffolds. Information was missing for *CmMLO9,* which could not be anchored onto a specific genomic position. (PNG 69 kb) Additional file 2: Figure S2. Reconstruction of *MLO* gene duplication events in *C. pepo* (A)*, C. lanatus* (B) and *C. melo* (C). Homologs with the highest value of pairwise identity (49.8 %, between CmMLO4 and CmMLO6) are boxed in red. The tree shows bootstrap values only when > 50. For each tree, the highest value of nucleotide identity for monophyletic pairs is indicated. (PNG 671 kb) Additional file 3: Figure S3. Identification of putative Cucurbitaceae *MLO* orthologs in synthenic genomic regions. Genomic regions of *C. lanatus*, *C. melo* and *C. pepo* include 20 kb upstream and downstream to *MLO* loci. Collinear blocks are labelled with the same colour and connected by lines. Block boundaries indicate breakpoints of genome rearrangements. Genomic alignment of putative *MLO* orthologs (OG2, OG3) in *C. lanatus*, *C. melo* and *C. pepo* synthenic regions (chromosomes or scaffolds). (PNG 229 kb) Additional file 4: Figure S4. Identification of putative Cucurbitaceae *MLO* orthologs in synthenic genomic regions. Genomic regions of *C. lanatus*, *C. melo* and *C. pepo* include 20 kb upstream and downstream to *MLO* loci. Collinear blocks are labelled with the same colour and connected by lines. Block boundaries indicate breakpoints of genome rearrangements Genomic alignment of putative *MLO* orthologs (OG5, OG7) in *C. lanatus*, *C. melo* and *C. pepo* synthenic regions (chromosomes or scaffolds). (PNG 603 kb) Additional file 5: Figure S5. Identification of putative Cucurbitaceae *MLO* orthologs in synthenic genomic regions. Genomic regions of *C. lanatus*, *C. melo* and *C. pepo* include 20 kb upstream and downstream to *MLO* loci. Collinear blocks are labelled with the same colour and connected by lines. Block boundaries indicate breakpoints of genome rearrangements. Genomic alignment of putative *MLO* orthologs (OP1, OP2) in *C. lanatus*, *C. melo* and *C. pepo* synthenic regions (chromosomes or scaffolds). (PNG 111 kb) Additional file 6: Figure S6. Identification of putative Cucurbitaceae *MLO* orthologs in synthenic genomic regions. Genomic regions of *C. lanatus*, *C. melo* and *C. pepo* include 20 kb upstream and downstream to *MLO* loci. Collinear blocks are labelled with the same colour and connected by lines. Block boundaries indicate breakpoints of genome rearrangements. Genomic alignment of putative MLO orthologs (OP3, OP4) in *C. lanatus*, *C. melo* and *C. pepo* synthenic regions (chromosomes or scaffolds). (PNG 183 kb) Additional file 7: Figure S7. Multiple alignment of MLO proteins. The dataset includes the 48 Cucurbitaceae MLO homologs identified in this study and 12 clade IV and clade V MLO homologs shown to play a role in powdery mildew susceptibility (barley HvMLO, rice OsMLO3, wheat TaMLO_B1 and TaMLO_A1b, Arabidopsis AtMLO2, AtMLO6 and AtMLO12, tomato SlMLO1, pepper CaMLO2, tobacco NtMLO1, pea PsMLO1, lotus LjMLO1 and barrel clover MtMLO1). Conserved region in MLO proteins. Green colour indicates 30 residues conserved (>95 %) throughout the whole MLO family, previously indicated as invariable from Elliot et al. (2005) [30]; blue indicates 13 residues conserved (>95 %) throughout the whole MLO family, identified in this study; red colour indicates 125 residues conserved (>90 %) in clade V Cucurbitaceae homologs and powdery mildew susceptibility factors from other species. Moreover, 25 patterns of consecutive conserved amino acids (motifs), distributed along all the known domains of MLO proteins and ranging in size from 2 to 11 residues are boxed. The star indicates position 252 of the alignment, predicted to be under positive selection by codon-based evolutionary analysis. (PNG 23839 kb) Additional file 8: Figure S8. Genomic localization of *CmMLO* and *ClMLO* homologs with respect to melon/watermelon synthenic regions. The figure is a modification of the one reported by Guo et al. 2013 [28], connecting macrosyntenic regions with colored lines. Black lines, connect eleven pairs of putative *MLO* orthologs identified in this study. (PNG 1175 kb) Additional file 9: Table S1. Gene-specific primers used in this study for gene-expression analysis. (XLSX 9 kb)

## Abbreviations

CRISPR: clustered regularly interspaced short palindromic repeat
GPCRs: G-protein coupled receptors
GUI: graphical user interface
HPI: hours post-inoculation
LCBs: locally collinear blocks
MAUVE: Multiple Alignment of Conserved Genomic Sequence with Rearrangements
MLO: Mildew locus O
MUSCLE: multiple sequence comparison by log- expectation
OG: ortholog groups
OP: ortholog pairs
PM: powdery mildew
R-genes: resistance genes
RNAi: RNA interference
SLAC: single likelihood ancestor counting
SNP: single nucleotide polymorphism
TALENs: transcription activator-like effector nucleases
TILLING: targeted induced local lesions in genomes
ZFNs: zinc finger nucleases
