# Supplementary figures and images for: Structure, evolution and functional inference on the Mildew Locus O (MLO) gene family in three cultivated Cucurbitaceae spp
Source: BMC Genomics. 2015 Dec 29;16:1112. doi: 10.1186/s12864-015-2325-3 (PMC4696115; doi:10.1186/s12864-015-2325-3)

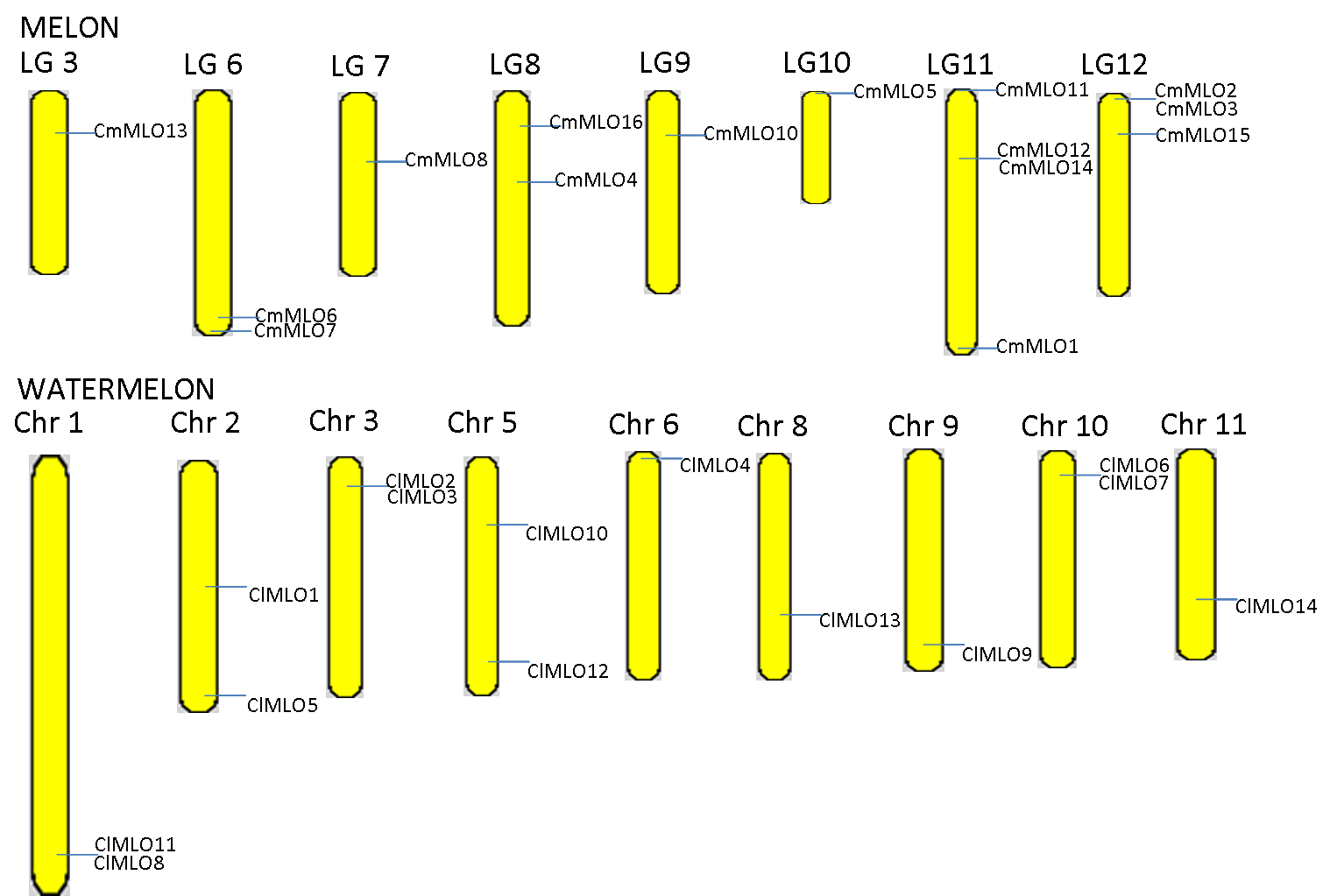

Supplement: Additional file 1: Figure S1. — Chromosomal localization of C. melo and C. lanatus MLO genes. Positions are estimated based on available information on physical localization on melon linkage groups or watermelon scaffolds. Information was missing for CmMLO9, which could not be anchored onto a specific genomic position. (PNG 69 kb) [file 12864_2015_2325_MOESM1_ESM.png]

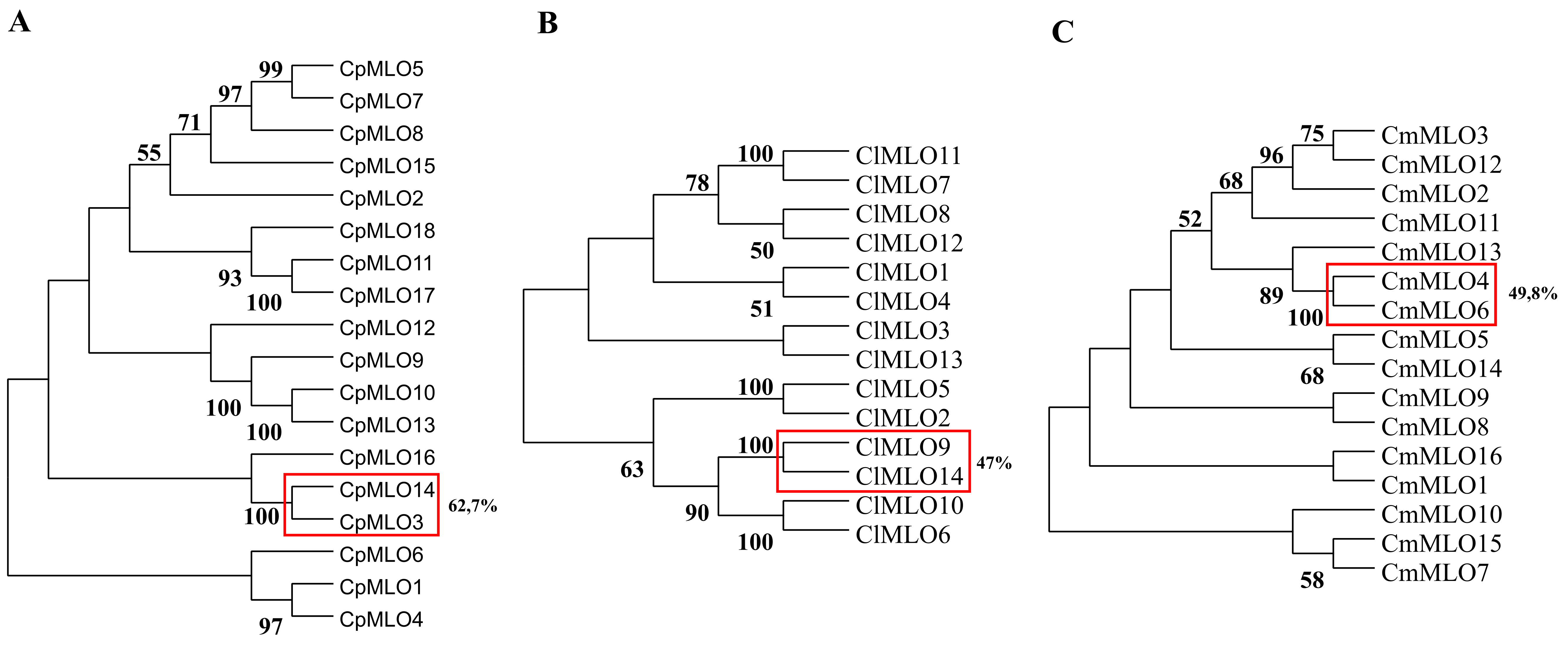

Supplement: Additional file 2: Figure S2. — Reconstruction of MLO gene duplication events in C. pepo (A), C. lanatus (B) and C. melo (C). Homologs with the highest value of pairwise identity (49.8 %, between CmMLO4 and CmMLO6) are boxed in red. The tree shows bootstrap values only when > 50. For each tree, the highest value of nucleotide identity for monophyletic pairs is indicated. (PNG 671 kb) [file 12864_2015_2325_MOESM2_ESM.png]

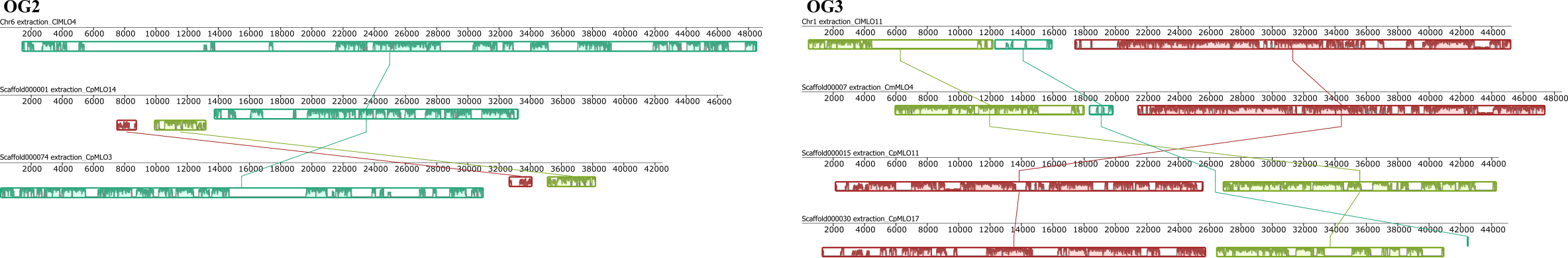

Supplement: Additional file 3: Figure S3. — Identification of putative Cucurbitaceae MLO orthologs in synthenic genomic regions. Genomic regions of C. lanatus, C. melo and C. pepo include 20 kb upstream and downstream to MLO loci. Collinear blocks are labelled with the same colour and connected by lines. Block boundaries indicate breakpoints of genome rearrangements. Genomic alignment of putative MLO orthologs (OG2, OG3) in C. lanatus, C. melo and C. pepo synthenic regions (chromosomes or scaffolds). (PNG 229 kb) [file 12864_2015_2325_MOESM3_ESM.png]

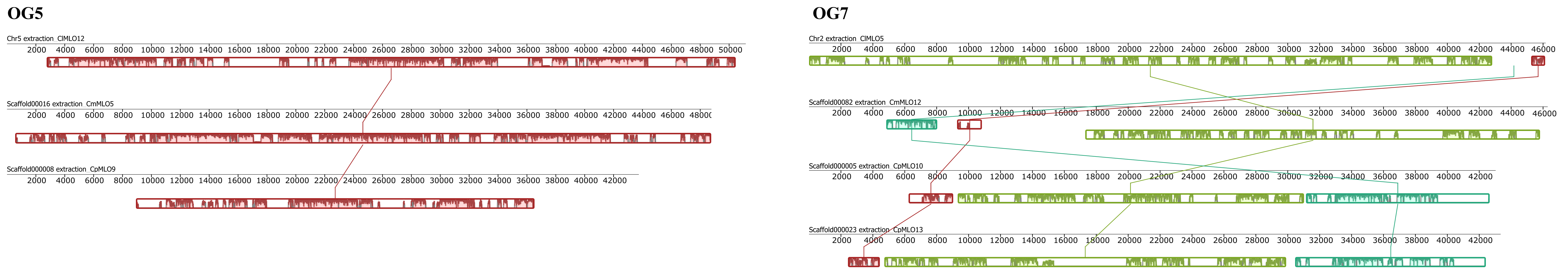

Supplement: Additional file 4: Figure S4. — Identification of putative Cucurbitaceae MLO orthologs in synthenic genomic regions. Genomic regions of C. lanatus, C. melo and C. pepo include 20 kb upstream and downstream to MLO loci. Collinear blocks are labelled with the same colour and connected by lines. Block boundaries indicate breakpoints of genome rearrangements Genomic alignment of putative MLO orthologs (OG5, OG7) in C. lanatus, C. melo and C. pepo synthenic regions (chromosomes or scaffolds). (PNG 603 kb) [file 12864_2015_2325_MOESM4_ESM.png]

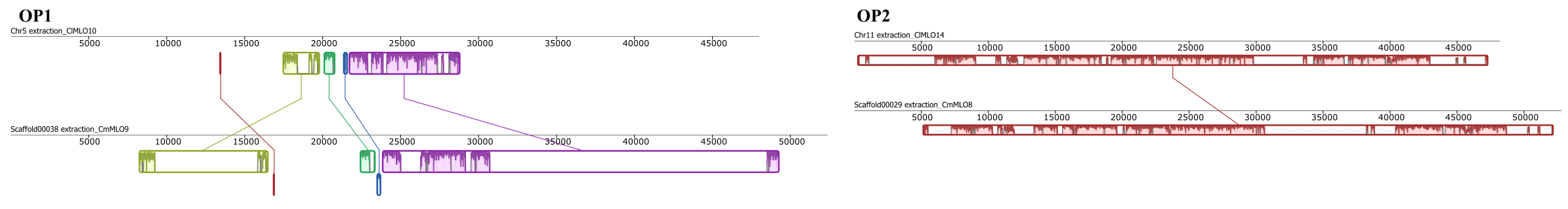

Supplement: Additional file 5: Figure S5. — Identification of putative Cucurbitaceae MLO orthologs in synthenic genomic regions. Genomic regions of C. lanatus, C. melo and C. pepo include 20 kb upstream and downstream to MLO loci. Collinear blocks are labelled with the same colour and connected by lines. Block boundaries indicate breakpoints of genome rearrangements. Genomic alignment of putative MLO orthologs (OP1, OP2) in C. lanatus, C. melo and C. pepo synthenic regions (chromosomes or scaffolds). (PNG 111 kb) [file 12864_2015_2325_MOESM5_ESM.png]

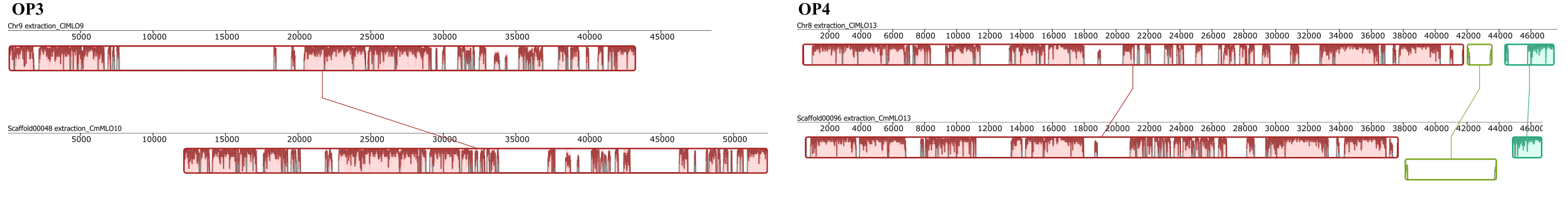

Supplement: Additional file 6: Figure S6. — Identification of putative Cucurbitaceae MLO orthologs in synthenic genomic regions. Genomic regions of C. lanatus, C. melo and C. pepo include 20 kb upstream and downstream to MLO loci. Collinear blocks are labelled with the same colour and connected by lines. Block boundaries indicate breakpoints of genome rearrangements. Genomic alignment of putative MLO orthologs (OP3, OP4) in C. lanatus, C. melo and C. pepo synthenic regions (chromosomes or scaffolds). (PNG 183 kb) [file 12864_2015_2325_MOESM6_ESM.png]

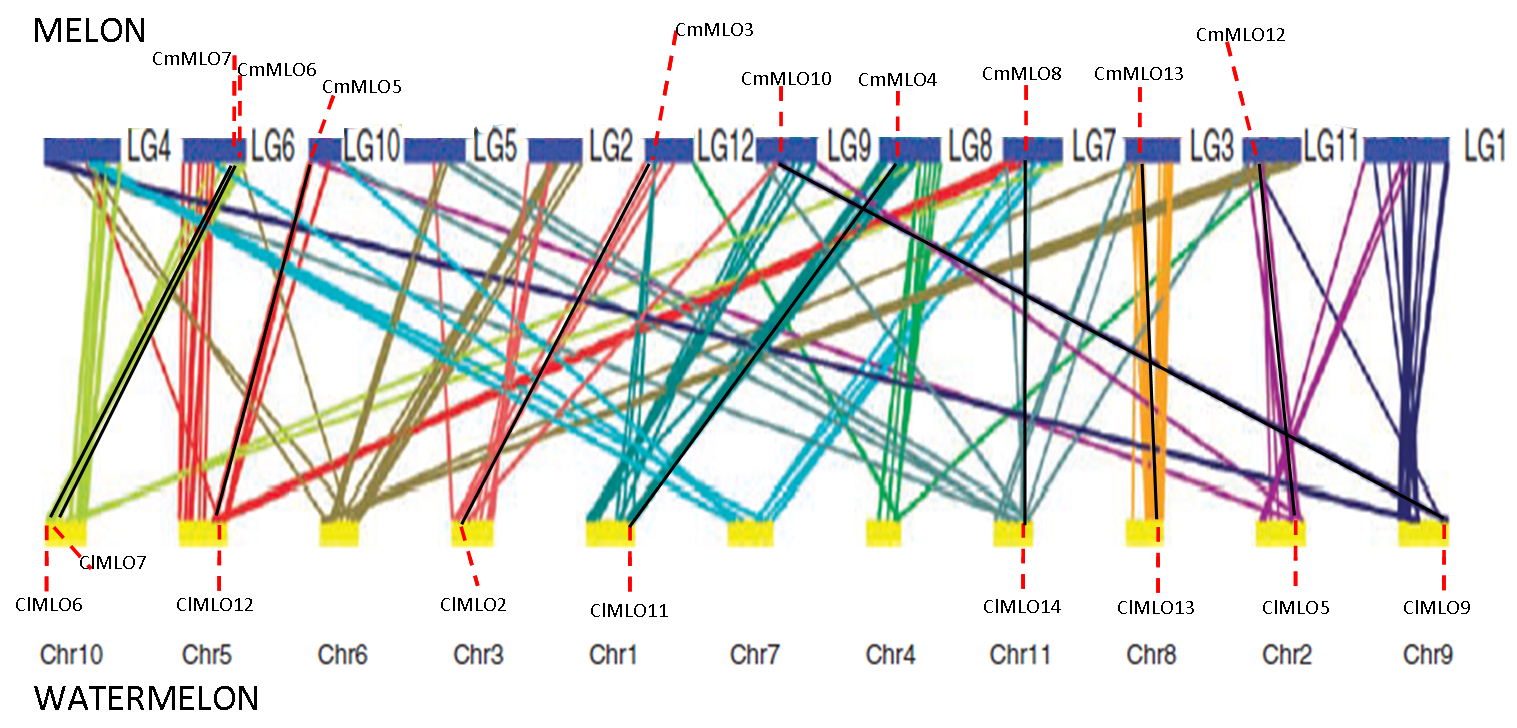

Supplement: Additional file 8: Figure S8. — Genomic localization of CmMLO and ClMLO homologs with respect to melon/watermelon synthenic regions. The figure is a modification of the one reported by Guo et al. 2013 [28], connecting macrosyntenic regions with colored lines. Black lines, connect eleven pairs of putative MLO orthologs identified in this study. (PNG 1175 kb) [file 12864_2015_2325_MOESM8_ESM.png]
